# Mean Daily Dosage of Aspirin and the Risk of Incident Alzheimer's Dementia in Patients with Type 2 Diabetes Mellitus: A Nationwide Retrospective Cohort Study in Taiwan

**DOI:** 10.1155/2016/9027484

**Published:** 2016-10-27

**Authors:** Cheng-Wei Chang, Jorng-Tzong Horng, Chi-Chang Hsu, Jui-Ming Chen

**Affiliations:** ^1^Department of Endocrinology and Metabolism, Tungs' Taichung MetroHarbor Hospital, Taichung 435, Taiwan; ^2^Department of Information Management, Hsing Wu University, New Taipei City, Taiwan; ^3^Department of Biomedical Informatics, Asia University, Taichung 413, Taiwan; ^4^Department of Computer Science and Information Engineering, National Central University, Chungli, Taiwan

## Abstract

*Background*. Type 2 diabetes mellitus patients are known to have higher risk of developing dementia while aspirin use has been shown to prevent incident dementia. This study was conducted to evaluate the potential benefits of aspirin use on dementia in patients with type 2 diabetes mellitus and identify the appropriate dosage of aspirin that provides the most benefit.* Method*. A Taiwan nationwide, population-based retrospective 8-year study was employed to analyze the association between the use of aspirin and incidence of dementia including Alzheimer's disease and non-Alzheimer's dementia using multivariate Cox-proportional hazards regression model and adjusting for several potential confounders.* Results*. Regular aspirin use in mean daily dosage of within 40 mg was associated with a decreased risk of developing incident Alzheimer's dementia in patients with type 2 diabetes mellitus (adjusted HR of 0.51 with 95% CI of 0.27–0.97, *p* value 0.041).* Conclusion*. A mean daily dosage of aspirin use within 40 mg might decrease the risk of developing Alzheimer's disease in patients with type 2 diabetes mellitus.

## 1. Introduction

Diabetic patients have been reported to have a greater decline in cognitive function and a higher risk of developing dementia, although some studies announced that the relation between diabetes and dementia remains contestable [1–6].

Many previous studies have indicated that patients taking NSAIDs or aspirin had a decreased risk of Alzheimer's disease [7–15], including case control, prospective, and meta-analytical studies. However, there are also other investigations that failed to confirm such protective effect of NSAIDs or aspirin [16–18]. One study indicated that high but not low dose aspirin can prevent Alzheimer's disease [15], while another case control study reported that such a dosage effect did not exist [9]. These controversial findings indicated the need for further research on whether the use of aspirin might benefit certain group of elderly subjects and the appropriate dose to be prescribed.

Although many previous studies revealed a positive association between aspirin use and dementia, few of them focused on type 2 diabetes mellitus patients and did not indicate the appropriate dose needed to prevent Alzheimer's disease. So, we conducted this study using the National Health Insurance Research Database (NHIRD) in Taiwan [19] to evaluate the effect of aspirin use on decreasing the risk of dementia in type 2 diabetes mellitus cohort and the appropriate dose of aspirin.

## 2. Materials and Method

### 2.1. Data Sources

This study utilized claims data from the National Health Insurance Research Dataset (NHIRD), maintained by the National Health Research Institute (NHRI) in Taiwan, which includes statistical information of more than 96% of the Taiwan population [20]. The National Health Insurance Research Database (NHIRD), which is provided to researchers for academic research purpose in Taiwan, contains a number of large computerized databases including original files and registration data. These data were obtained from the insurance system constructed by the Bureau of National Health Insurance (NHI). These files are deidentified by scrambling the identification codes of individuals and medical facilities; then the patients' information is sent to the National Health Research Institutes and forms the original files of the NHIRD [21].

This study used a 1,000,000-individual randomly selected sample from the Taiwan NHIRD. The enrolled patients were followed up from 1997 to 2008.

In this article, we applied the databases for patients' information including encoded identification number, gender, birth and death date, diagnostic data, and procedures and discharge status (diagnosed cancers before, died or transferred out, less than three diagnoses). The diagnostic data included initial diagnosis date, specific treatment items, and the relevant International Classification of Diseases, Revision Ninth, Clinical Modification (ICD-9-CM) diagnosis codes.

### 2.2. Study Sample

This study was designed as a population-based retrospective cohort study. Cohort study is an association research study that tracks the same people over a period of time for identifying the incidence of dementia occurrence with or without exposure to the use of aspirin. The study sample was drawn from 1997–2008 Taiwan NHIRD [19]. As the data files consisted of unidentified secondary data (National Health Insurance Research Database), the study was exempted from a full review by the Institutional Review Board of Tungs' Taichung MetroHarbor Hospital. Obtaining informed consent from the study population was not required due to the deidentified data files, the large size of the population (1 million), and the deceased status of some of the population by the time of the study.

**Figure 1:**
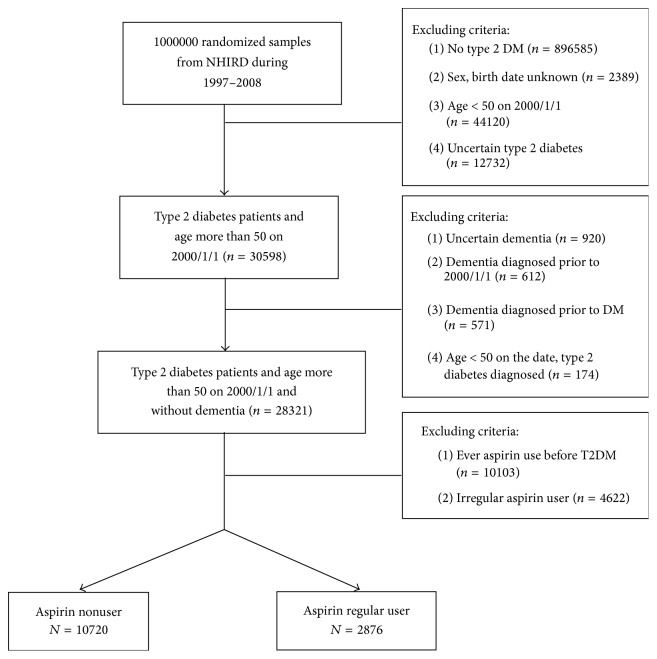
Study flowchart.

We identified 28,321 patients with diagnosis of type 2 diabetes mellitus who were above 50 years old with no history of dementia on January 1, 2000, and who presented in ambulatory visit file for at least 2 times within any one year between 2000 and 2008. NHIRD employed A-code until December 31, 1999. Since A-code cannot precisely identify subjects with Alzheimer's disease and non-Alzheimer dementia, we excluded type 2 diabetes mellitus patients with dementia (*n* = 612) (A-codes A210 and A222) before 2000 from the study samples. The study then separated the type 2 diabetes mellitus samples into two groups: those who had never used aspirin (*n* = 10,720) and those who used aspirin regularly (*n* = 2,876). The detailed study flow was presented in Figure 1.

We defined regular aspirin users as the regular use of aspirin for more than a year and the interval between successive prescription drug records cannot exceed 120 days. A total of 13,596 patients in this study were individually traced until December 31, 2008, after index prescription of aspirin to identify patients who had been diagnosed with Alzheimer's disease (ICD-9-CM codes 290.0, 290.10–290.13, 290.20, 290.21, 290.3, 294.1, and 331.0) or non-Alzheimer dementia (ICD-9-CM codes 046.1, 290.1, 290.2, 290.4, 290.40–290.43, 294.11, 331.1, 331.11, 331.19, 331.2, and 331.7–331.9) at least twice in any one year during the follow-up period. In this study, we further classified regular aspirin users into three subgroups based on their average daily dose.

The index date of follow-up period for the group that never used aspirin was assigned to the date of type 2 diabetes mellitus diagnosis, whereas the index date of follow-up period for the group that used aspirin regularly was assigned to the first prescription date of aspirin. The end date of follow-up period for both nonaspirin users and regular aspirin users was assigned to the date of dementia, the date of death, or 31 of December, 2008, whichever came first. The dose of aspirin used was a mean daily dose which was calculated by cumulative doses divided by cumulative observation days. It is not the real daily dose prescribed to the patients.

The comorbid medical conditions for each individual were evaluated by using the established Charlson-Deyo comorbidity index (CCI). Chronic concomitant diseases of study samples related to arthropathy, cardiovascular, gastrointestinal, hepatic, neoplastic, neurologic, pulmonary, and renal diseases were categorized from the CCI index and have been described in detail in Table 1.

### 2.3. Statistical Analysis

The SAS statistical software (version 9.2) was used to perform all programming and statistical analyses in this study. The *t*-test and Pearson *χ*2 test were used to examine the differences in demographic characteristics of T2DM patients with and without regular use of aspirin. Demographic characteristics included categorical variables such as age group, gender, types of stroke, statins, antihypertensive drugs, CCI group, follow-up group, antidiabetic drug types, and underlying chronic diseases.

The risk for dementia associated with type 2 diabetes mellitus and exposure to regular use of aspirin was estimated by the cox proportional hazards models. Cox proportional hazards models provided both unadjusted and adjusted hazard ratios with 95% confidence interval. The adjusted hazard ratios included age group, gender, CCI group, stroke types, and antidiabetic drugs as potential covariates. The Kaplan-Meier method was used to compare the cumulative Alzheimer's disease and non-Alzheimer dementia events-free unjustified survival probabilities from 1997 to 2008 with different dosage of aspirin used in patients with type 2 diabetes mellitus. Survival curves among type 2 diabetes mellitus patients were created individually based on their aspirin medication status. The log-rank test was used to determine the significance of inequality with respect to aspirin medication status curves.

## 3. Results

### 3.1. Demographic Characteristics

A total of 28,321 patients diagnosed with type 2 diabetes mellitus whose age was above 50 and dementia-free before 1 January 2000 were selected. Among these, 10,720 were patients who never used aspirin and 2,876 were defined as patients who regularly used aspirin. Table 1 compared the baseline characteristics between the aspirin users group and nonaspirin users group. Aspirin users with type 2 diabetes mellitus were older and more often male, more often had history of stroke, used more types of antidiabetes drugs, used antihypertensive drugs and statins more often, and had more chronic comorbidities. The mean duration of follow-up for the aspirin users group was 4.5 years and 5.0 years in nonaspirin users group.

#### 3.1.1. Kaplan-Meier Dementia-Free Survival Curves

**Figure 2:**
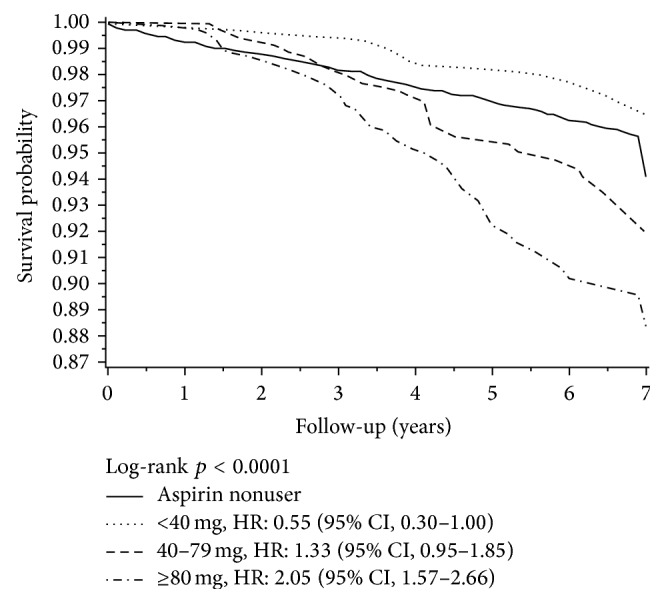
All-cause dementia-free survival curves by mean daily dosage of aspirin.

The log-rank test indicated that the nonaspirin users had significantly higher Alzheimer's disease and non-Alzheimer dementia events-free unjustified survival probabilities than aspirin users in Supplemental Figures 1 and 2 (see Supplementary Material available online at http://dx.doi.org/10.1155/2016/9027484). It is interesting to note that aspirin users had lower Alzheimer's disease and non-Alzheimer dementia events-free survival probabilities not until 2.5 years of follow-up and 1.5 years of follow-up, respectively. The regular aspirin users were then further classified into three subgroups based on their average daily dose. In Figure 2 and Supplemental Figures 3 and 4, regular aspirin users with low mean daily dose of aspirin (<40 mg) had higher Alzheimer's disease and non-Alzheimer's dementia events-free unjustified survival probabilities than the other subgroups and nonaspirin users.

#### 3.1.2. Risk of Alzheimer's Disease and Non-Alzheimer's Dementia

Table 2 presents the results of the cox proportional hazard model for different status of aspirin usage during the follow-up period. Type 2 diabetes mellitus patients using aspirin had a higher risk of Alzheimer's disease (unadjusted HR: 1.33; 95% CI 1.06–1.68, *p* = 0.016) and non-Alzheimer's dementia (unadjusted HR: 1.94; 95% CI 1.19–3.16, *p* = 0.008) than type 2 diabetes mellitus patients without using aspirin in the unadjusted analyses. After adjusting for age, gender, history of stroke, types of antidiabetic drugs, statins, antihypertensive drugs, and CCI score, the adjusted results revealed no significant differences in Alzheimer's disease (adjusted HR: 1.37; 95% CI 1.05–1.78, *p* = 0.019) and non-Alzheimer's dementia (adjusted HR: 1.91; 95% CI 1.09–3.36, *p* = 0.025) between type 2 diabetes mellitus patients with or without use of aspirin.

For the subgroup prescribed with a mean daily dose of less than 40 mg of aspirin, there was a trend towards a reduced risk of Alzheimer's disease (adjusted HR: 0.51, 95% CI 0.27–0.97, *p* value 0.041) as compared with nonaspirin users. However, no trend was detected in the risk of non-Alzheimer's dementia (adjusted HR: 0.28; 95% CI 0.04–2.05, *p* = 0.209) for patients prescribed with a mean daily dose of less than 40 mg of aspirin. In the subgroup prescribed with a mean daily dose between 40 mg and 80 mg of aspirin, the adjusted ratios were 1.27 (95% CI 0.84–1.91) and 2.54 (95% CI 1.23–5.24) for the risk of Alzheimer's disease and non-Alzheimer's dementia, respectively. For the subgroup prescribed with a mean daily dose of more than 80 mg of aspirin, an increased risk of developing Alzheimer's disease (adjusted HR: 2.26, 95% CI 1.64–3.12, *p* < 0.001) and non-Alzheimer's dementia (adjusted HR: 2.95, 95% CI 1.44–6.05, *p* = 0.003) was found in this study. As can be seen from Table 2, the risk of non-Alzheimer's dementia in type 2 diabetes mellitus patients increased with the increasing amount of aspirin usage.

## 4. Discussion

The main finding of this study showed that regular use of aspirin in a mean daily dose of within 40 mg might decrease the risk of developing Alzheimer's disease in patients with type 2 diabetes mellitus while no benefit was observed in non-Alzheimer's dementia. But once the mean daily dose of aspirin was higher than 80 mg per day, both the risks of incident Alzheimer's dementia and non-Alzheimer's dementia increased in patients with type 2 diabetes mellitus.

Our data confirmed the association between aspirin use and risk reduction of Alzheimer's dementia as seen in previous reports [7, 9, 10, 14, 15, 22–24]. In 2000, Broe et al. reported an inverse association between aspirin and Alzheimer's dementia, but such association was not observed with vascular dementia [9]. Another population study of Alzheimer's dementia in Cache County reported that use of aspirin was also specifically associated with reduced occurrence of Alzheimer's dementia [11]. But the above studies did not specifically target type 2 diabetes mellitus patients and their patient numbers were also relatively smaller compared to our study. Besides, most of them also did not indicate the appropriate dose of aspirin used to prevent Alzheimer dementia. Their analysis also did not distinguish Alzheimer dementia and non-Alzheimer dementia.

There are also some studies with opposing results. An investigation of up to 2,300 participants from the Baltimore Longitudinal Study of Aging concluded that aspirin use was associated with greater prospective cognitive decline on select measures [16]. Another 12-year longitudinal clinical-pathologic study of 1,019 older Catholic clergy did not support a strong relation between aspirin and Alzheimer disease (HR 0.84, 95% CI 0.63–1.11) [17]. Results from a population-based cross-sectional study with 2,708 patients enrolled revealed that long-term NSAIDs use has a protective effect against Alzheimer's dementia, but this association was statistically significant only for nonaspirin NSAIDs use [25]. Another two-wave longitudinal study over 3.6 years also was not able to conclude the protective effect of aspirin on Alzheimer dementia [18].

In our present study, we did not show a risk reduction of non-Alzheimer dementia in response to regular aspirin use. The lack of statistical significance of the protecting effects of low dose aspirin in developing non-Alzheimer dementia was likely due to limited sample size since the punctual estimate was quite low (HR = 0.28). In fact, vascular dementia accounts for about 20–30% of all the dementia and comprises the majority of non-Alzheimer disease dementia. In support of our findings, Broe et al. reported an inverse association between aspirin and Alzheimer's dementia, but such association was not observed with vascular dementia [9].

In our study, we also found that a mean daily dose of aspirin of within 40 mg is statistically significant in preventing Alzheimer's dementia (adjusted HR: 0.48, 95% CI 0.25–0.90, *p* value 0.022). Although there were many studies about the use of aspirin in preventing Alzheimer's dementia, only a few of them indicated the appropriate dose. A Swedish population-based study on individuals 80 years old or more revealed users of high-dose (>500 mg/day) aspirin had significantly lower prevalence of Alzheimer's dementia whereas users of 75 mg daily dose had only numerical but insignificant reduction of Alzheimer's dementia, even after correction of stroke, transient ischemic attack, myocardial infarction, angina pectoris, and congestive heart failure [15]. In some studies examining dosage effects, elderly persons who took high-dose NSAIDs got poorer memory and decline faster than those taking low doses [26]. Another case control study involving subjects with average age of 81 disclosed that not only a high-dose (>1000 mg/day) anti-inflammatory action of aspirin but also low-dose antiplatelet action (<500 mg/day) is protective against Alzheimer's dementia [9]. Their results suggested that the anti-inflammatory drug hypothesis of Alzheimer's dementia prevention should be reviewed. In addition, alternate mechanisms of low-dose NSAID and/or aspirin drug action should be considered. They proposed that such low doses of NSAID and/or aspirin can protect against Alzheimer's dementia by ameliorating platelet and endothelium dysfunction [9].

As with most retrospective studies, there are strengths and limitations in our study. The greatest strength of our study lies in its large-scale population-based data and relative longer follow-up period (8 years). But there are some pertinent limitations in our study. The dose of aspirin used was a mean daily dose which was calculated by cumulative dose divided by cumulative observation days. It was not the real daily dose prescribed to the patients.

Besides, we only identified 11 patients with all-cause dementia whose mean daily dose of aspirin was less than 40 mg. Therefore, if we further divided those 11 patients into 4 different age groups, a relative low sample size has a weak power to detect the difference in significance which is listed in Table 2. Also, ICD-9 codes may not accurately reflect the patients' diagnoses due to some artificial errors. Besides, the clinical status of patients' glycemic and blood pressure control, other potential risk factors for dementia such as education, diet, smoking, and alcohol use and apolipoprotein E4 genotype were not provided by the administrative claims dataset [6]. Another limitation is that our study cohort is all Taiwanese people; the results of our study may not apply to type 2 diabetic patients with different ethnicity.

## 5. Conclusion

Mean daily dose of aspirin use within 40 mg might decrease the risk of developing Alzheimer's disease in patients with type 2 diabetes mellitus while no benefit was observed in non-Alzheimer's dementia. But once the mean daily dose of aspirin was higher than 80 mg per day, both the risks of incident Alzheimer's dementia and non-Alzheimer's dementia increased in patients with type 2 diabetes mellitus. The exact mechanism of these effects needs further elucidation and investigations.

## Supplementary Material

Supplemental Figure 1: All-cause dementia free survival curves
Supplemental Figure 2: Alzheimer's disease free survival curves
Supplemental Figure 3: Alzheimer's disease free survival curves by mean daily dosages of aspirin
Supplemental figure 4: Non-Alzheimer dementia free survival curves by mean daily dosages of aspirin

## Figures and Tables

**Table 1:** Demographics of study subjects by aspirin using conditions between 1997 and 2008 in Taiwan.

Descriptor	Nonaspirin users among T2DM patients^a^		Aspirin users among T2DM patients^b^		*p* value^c^
	10720	(%)	2876	(%)	
Age group∖mean ± SD	64.8 ± 8.2		66.9 ± 7.9		<0.001
50–59	3507	32.7	618	21.5	<0.001
60–69	4469	41.7	1265	44.0	
70–79	2182	20.4	810	28.2	
≥80	562	5.2	183	6.4	
Gender					
Male	5194	48.5	1479	51.4	<0.005
Female	5526	51.5	1397	48.6	
Stroke^d^	385	3.6	435	15.1	<0.001
No stroke	10335	96.4	2441	84.9	<0.001
Haemorrhagic stroke	123	1.1	49	1.7	<0.018
Ischemic stroke	154	1.4	262	9.1	<0.001
Transient ischemic stroke	69	0.6	87	3.0	<0.001
Unclassified	93	0.9	113	3.9	<0.001
Antidiabetes drug type^e^					
Never use	7026	65.5	880	30.6	<0.001
Acarbose	357	3.3	292	10.2	<0.001
Metformin	2291	21.4	1308	45.5	<0.001
Thiazolidinedione (TZD)	196	1.8	141	4.9	<0.001
Sulfonylureas	2563	23.9	1481	51.5	<0.001
Meglitinide	246	2.3	193	6.7	<0.001
Insulin	101	0.9	111	3.9	<0.001
Statin type^e^					
Never use	9811	91.5	2193	76.3	<0.001
Atorvastatin	352	3.3	296	10.3	<0.001
Fluvastatin	104	1.0	93	3.2	<0.001
Lovastatin	209	1.9	98	3.4	<0.001
Pravastatin	108	1.0	67	2.3	<0.001
Rosuvastatin	102	1.0	114	4.0	<0.001
Simvastatin	172	1.6	133	4.6	<0.001
Hypertensive drug type^e^					
Never use	6418	59.9	509	17.7	<0.001
Alpha blocker	545	5.1	294	10.2	<0.001
ARB	1415	13.2	1116	38.8	<0.001
ACEI	1073	10.0	755	26.3	<0.001
Beta blocker	1649	15.4	1087	37.8	<0.001
CCB	2560	23.9	1567	54.5	<0.001
Diuretics	785	7.3	498	17.3	<0.001
CCI score^f^ ∖ mean ± SD	1.1 ± 1.3		1.6 ± 1.5		<0.001
CCI score of 0	4375	40.8	782	27.2	<0.001
CCI score of 1, 2	4813	44.9	1465	50.9	
CCI score of 3, 4	1251	11.7	500	17.4	
CCI score of ≥5	281	2.6	129	4.5	
Chronic diseases^d^					
No chronic disease	4046	37.7	744	25.9	<0.001
Arthropathy	414	3.9	132	4.6	0.078
Cardiovascular	1480	13.8	750	26.1	<0.001
Gastrointestinal	1600	14.9	428	14.9	0.954
Hepatic	2358	22.0	873	30.4	<0.001
Neoplasm	313	2.9	97	3.4	0.208
Neurologic	46	0.4	22	0.8	0.024
Pulmonary	3820	35.6	1224	42.6	<0.001
Renal	1065	9.9	439	15.3	<0.001

^a^Diagnosed T2DM patients who did not use aspirin before the end of follow-up date.

^b^The aspirin regular user is the patient who uses aspirin continuously at least over one year in the follow-up time that nearly 2 aspirin prescriptions cannot exceed 120 days. This group of diagnosed T2DM patients is aspirin regular user group.

^c^Wilcoxon rank sum test and Pearson's Chi-square test.

^d^The case number is calculated before patient's index date. The index date of patient who never uses aspirin before the end of follow-up date is the T2DM diagnosed date. The index date of patient who is aspirin regular user is the date of starting to take aspirin regularly.

^e^The case number is the regular use of the specific drugs before patient's end date of observation.

^f^Charlson comorbidity index (CCI). The diagnoses recorded in the National Health Insurance Research Database before the index date are used to calculate CCI score. We exclude the diagnosis of diabetes mellitus and stroke from CCI score calculation, because these two disease entities are considered separately.

SD: standard deviation, T2DM: type 2 diabetes mellitus, ARB: angiotensin II receptor blockers, ACEI: angiotensin converting enzyme inhibitors, and CCB: calcium channel blocker.

**Table 2:** Risk of Alzheimer's disease, non-Alzheimer dementia, and all-cause dementia among T2DM patients who regularly use aspirin.

Study subjects		All-cause dementia				
Aspirin status	Mean daily dose (mg)^a^	Dementia cases/total	Unadjusted HR (95% CI)	*p* value	Adjusted HR (95% CI)^b^	*p* value
No aspirin		360/10720	Reference (N/A)		Reference (N/A)	
Regular user		117/2876	1.42 (1.15–1.75)	0.001	1.45 (1.15–1.84)	0.002
Cohort 1	<40	11/522	0.55 (0.30–1.00)	0.050	0.48 (0.26–0.89)	0.019
Cohort 2	40–79	39/1003	1.33 (0.95–1.85)	0.096	1.47 (1.03–2.09)	0.033
Cohort 3	≥80	67/1351	2.05 (1.57–2.66)	<0.001	2.35 (1.75–3.15)	<0.001

Study subjects		Alzheimer's disease				
Aspirin status	Mean daily dose (mg)^a^	Dementia cases/total	Unadjusted HR (95% CI)	*p* value	Adjusted HR (95% CI)^b^	*p* value

No aspirin		308/10668	Reference (N/A)		Reference (N/A)	
Regular user		93/2852	1.33 (1.06–1.68)	0.016	1.37 (1.05–1.78)	0.019
Cohort 1	<40	10/521	0.58 (0.31–1.10)	0.094	0.51 (0.27–0.97)	0.041
Cohort 2	40–79	28/992	1.13 (0.77–1.66)	0.545	1.27 (0.84–1.91)	0.257
Cohort 3	≥80	55/1339	1.99 (1.49–2.66)	<0.001	2.26 (1.64–3.12)	<0.001

Study subjects		Non-Alzheimer dementia				
Aspirin status	Mean daily dose (mg)^a^	Dementia cases/total	Unadjusted HR (95% CI)	*p* value	Adjusted HR (95% CI)^b^	*p* value

No aspirin		52/10412	Reference (N/A)		Reference (N/A)	
Regular user		24/2783	1.94 (1.19–3.16)	0.008	1.91 (1.09–3.36)	0.025
Cohort 1	<40	1/512	0.34 (0.05–2.46)	0.286	0.28 (0.04–2.05)	0.209
Cohort 2	40–79	11/975	2.49 (1.29–4.78)	0.006	2.54 (1.23–5.24)	0.012
Cohort 3	≥80	12/1296	2.42 (1.29–4.57)	0.006	2.95 (1.44–6.05)	0.003

^a^Mean daily dose (mg) = cumulative doses starting from the regular taking drug date to the end of observation date/days between the start regular taking drug date and the end of observation date.

^b^Adjust age group, gender, CCI group, stroke types, antidiabetic drugs, statins, and hypertensive drugs.

HR: hazard ratio; CI: confidence interval; T2DM: type 2 diabetes mellitus.
