# Supplementary material for: Mean Daily Dosage of Aspirin and the Risk of Incident Alzheimer's Dementia in Patients with Type 2 Diabetes Mellitus: A Nationwide Retrospective Cohort Study in Taiwan
Source: J Diabetes Res. 2016 Oct 27;2016:9027484. doi: 10.1155/2016/9027484 (PMC5102734; doi:10.1155/2016/9027484)
Supplement: Supplementary file 1 — Supplemental Figure 1: All-cause dementia free survival curves Supplemental Figure 2: Alzheimer's disease free survival curves Supplemental Figure 3: Alzheimer's disease free survival curves by mean daily dosages of aspirin Supplemental figure 4: Non-Alzheimer dementia free survival curves by mean daily dosages of aspirin [file 9027484.f1.pdf]

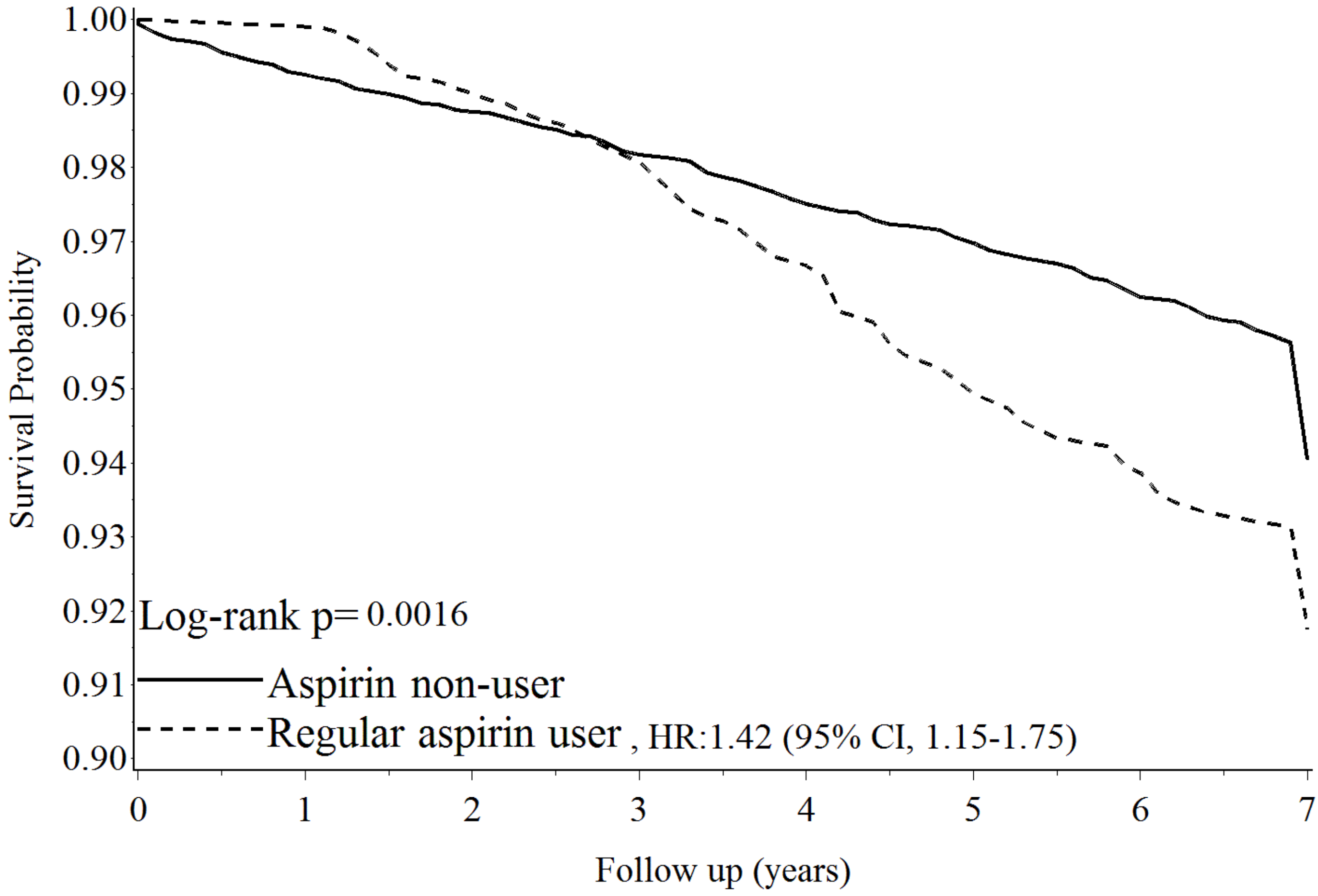

Supplemental Figure 1: All-cause dementia free survival curves

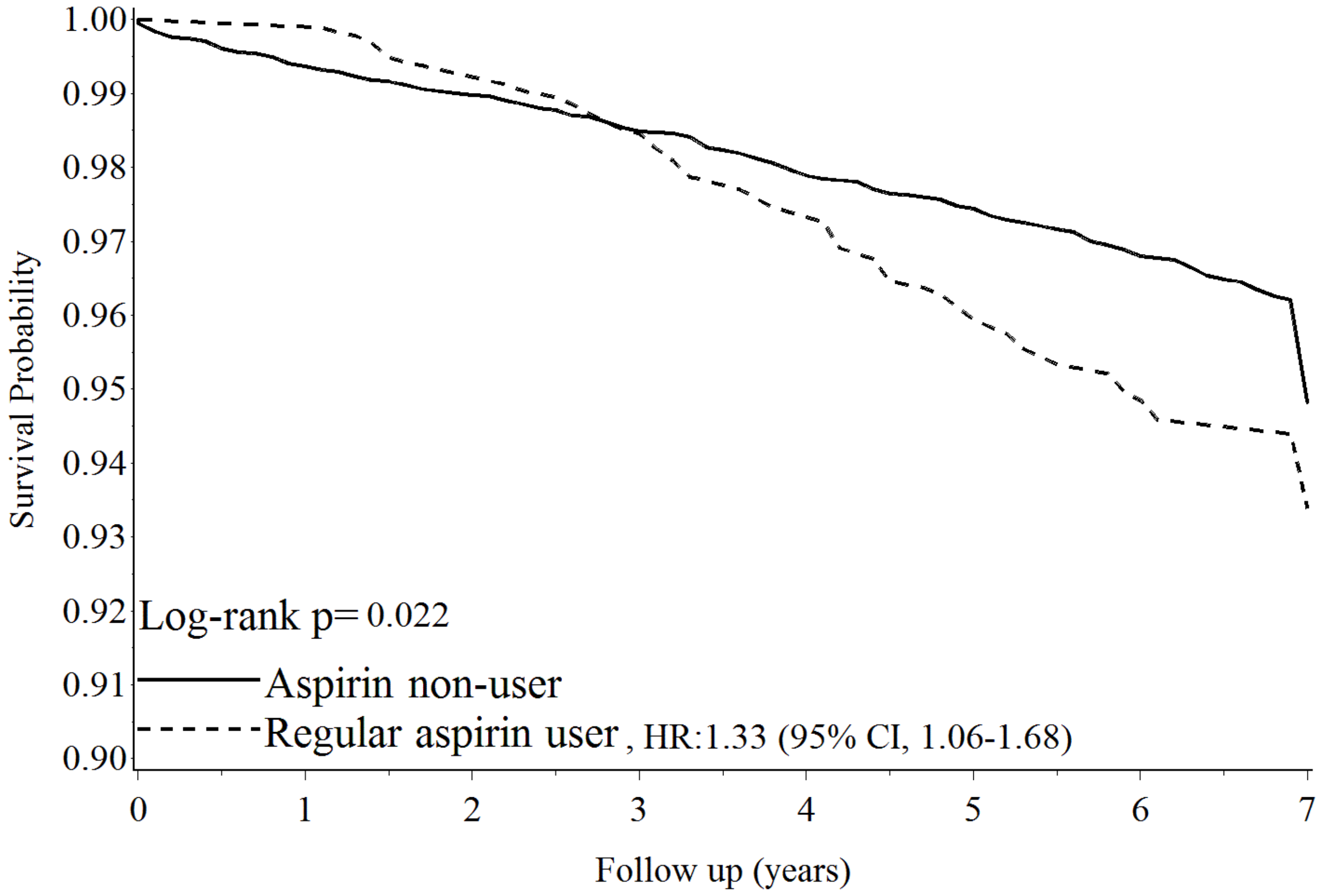

Supplemental Figure 2: Alzheimer's disease free survival curves

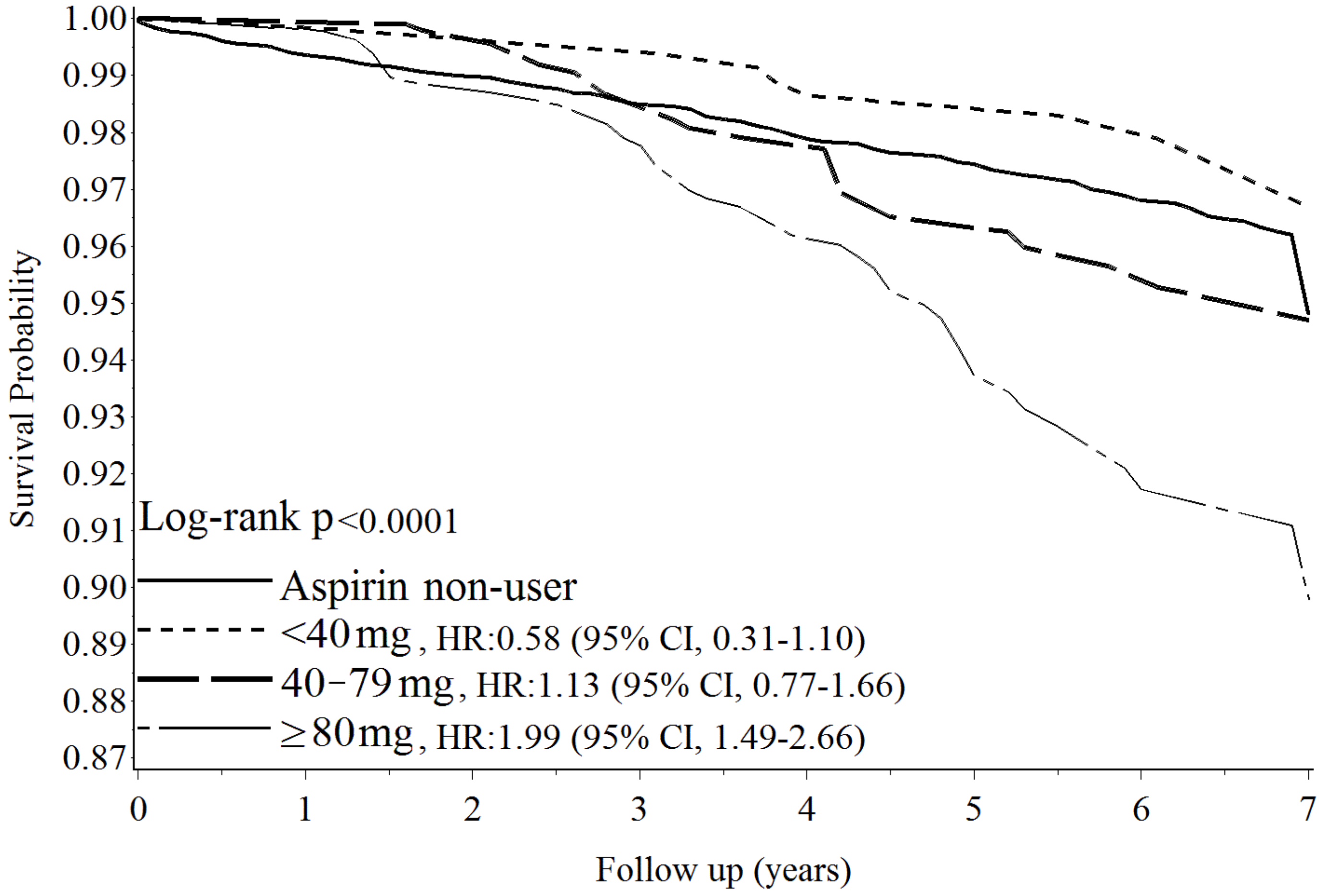

Supplemental Figure 3: Alzheimer's disease free survival curves by mean daily dosages of aspirin

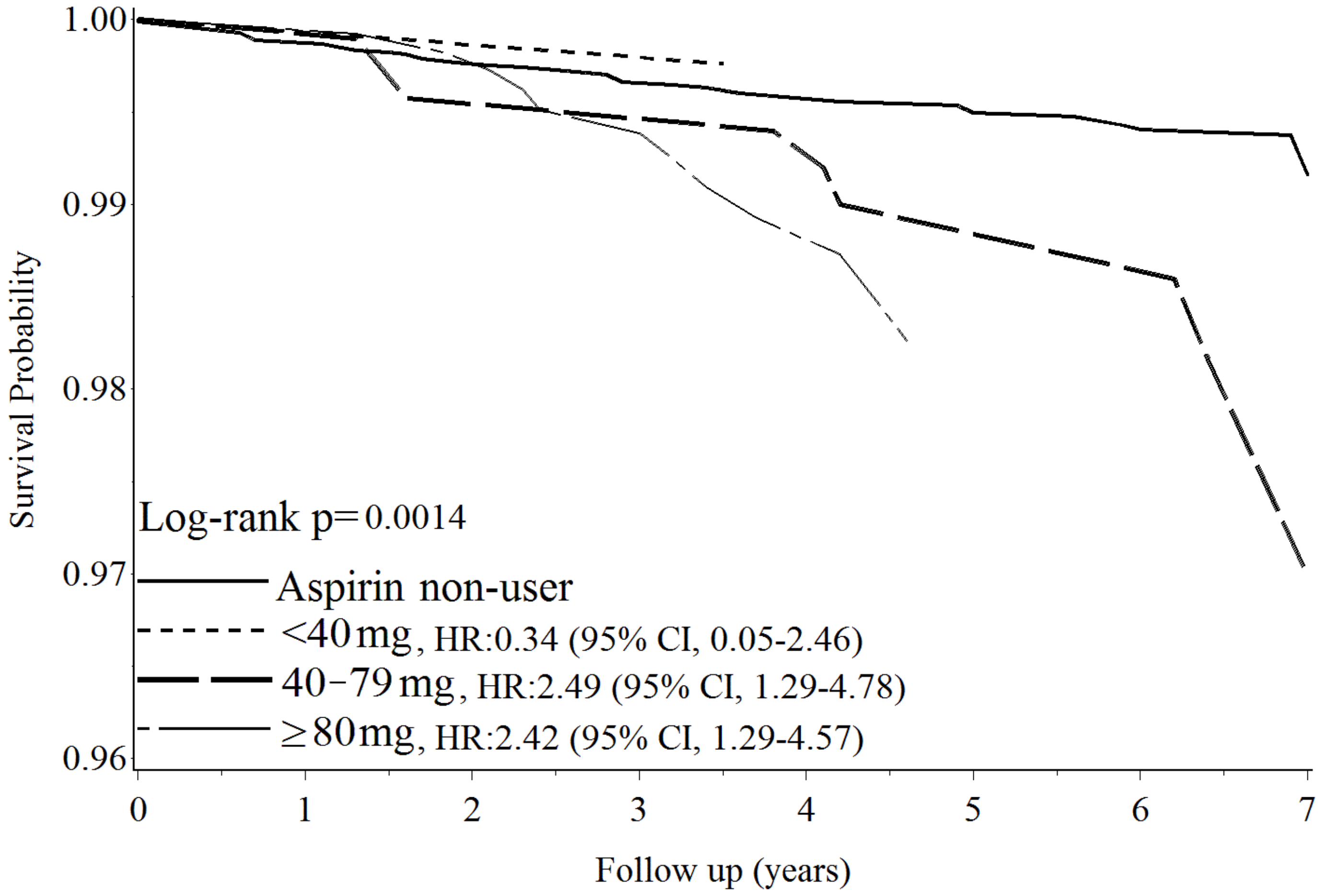

Supplemental figure 4: Non-Alzheimer dementia free survival curves by mean daily dosages of aspirin
